# A saturated SSR/DArT linkage map of *Musa acuminata *addressing genome rearrangements among bananas

**DOI:** 10.1186/1471-2229-10-65

**Published:** 2010-04-13

**Authors:** Isabelle Hippolyte, Frederic Bakry, Marc Seguin, Laetitia Gardes, Ronan Rivallan, Ange-Marie Risterucci, Christophe Jenny, Xavier Perrier, Françoise Carreel, Xavier Argout, Pietro Piffanelli, Imtiaz A Khan, Robert NG Miller, Georgios J Pappas, Didier Mbéguié-A-Mbéguié, Takashi Matsumoto, Veronique De Bernardinis, Eric Huttner, Andrzej Kilian, Franc-Christophe Baurens, Angélique D'Hont, François Cote, Brigitte Courtois, Jean-Christophe Glaszmann

**Affiliations:** 1 CIRAD, UR Multiplication Végétative, Av. Agropolis, 34398 Montpellier Cedex 5, France; 2 CIRAD, UMR DAP, Av. Agropolis, 34398 Montpellier Cedex 5, France; 3 Current address: CIRAD, UMR Contrôle des maladies, Campus Baillarguet, 34398 Montpellier Cedex 5, France; 4 CIRAD, UR Multiplication Végétative, Station de Neufchâteau, Sainte-Marie, 97130 Capesterre Belle-Eau, Guadeloupe FWI; 5 Current address: CIRAD, UMR BGPI, Campus international de Baillarguet, 34398 Montpellier Cedex 5, France; 6 Current address: Genomics Platform, Parco Tecnologico Padano, Via Einstein, Lodi, Italy; 7 Nuclear Institute of Agriculture, Tando Jam, Sindh, Pakistan; 8 Instituto de Ciências Biológicas, Departamento de Biologia Celular, Universidade de Brasília, Campus Universitário Darcy Ribeiro, Asa Norte, CEP 70.910-900, Brasília, Brazil; 9 Embrapa Recursos Genéticos e Biotecnologia, Brasília, Brazil; 10 UMR QUALITROP, Station de Neufchâteau, Sainte-Marie, 97130 Capesterre Belle-Eau, Guadeloupe FWI; 11 NIAS, Plant Genome Research Unit, Division of Genome and Biodiversity Research, 2-1-2, Kannondai, Tsukuba, Ibaraki 305-8602, Japan; 12 CEA, DSV, IG, Genoscope, 2 rue Gaston Crémieux, 91000 Evry, France; 13 Diversity Arrays Technology, PO Box 7141, Yarralumla, ACT 2600, Australia; 14 CIRAD, UPR Systèmes bananes et ananas, Boulevard de la Lironde, 34398 Montpellier Cedex 5, France

## Abstract

**Background:**

The genus *Musa *is a large species complex which includes cultivars at diploid and triploid levels. These sterile and vegetatively propagated cultivars are based on the A genome from *Musa acuminata*, exclusively for sweet bananas such as Cavendish, or associated with the B genome (*Musa balbisiana*) in cooking bananas such as Plantain varieties. In *M. acuminata *cultivars, structural heterozygosity is thought to be one of the main causes of sterility, which is essential for obtaining seedless fruits but hampers breeding. Only partial genetic maps are presently available due to chromosomal rearrangements within the parents of the mapping populations. This causes large segregation distortions inducing pseudo-linkages and difficulties in ordering markers in the linkage groups. The present study aims at producing a saturated linkage map of *M. acuminata*, taking into account hypotheses on the structural heterozygosity of the parents.

**Results:**

An F_1 _progeny of 180 individuals was obtained from a cross between two genetically distant accessions of *M. acuminata*, 'Borneo' and 'Pisang Lilin' (P. Lilin). Based on the gametic recombination of each parent, two parental maps composed of SSR and DArT markers were established. A significant proportion of the markers (21.7%) deviated (p < 0.05) from the expected Mendelian ratios. These skewed markers were distributed in different linkage groups for each parent. To solve some complex ordering of the markers on linkage groups, we associated tools such as tree-like graphic representations, recombination frequency statistics and cytogenetical studies to identify structural rearrangements and build parsimonious linkage group order. An illustration of such an approach is given for the P. Lilin parent.

**Conclusions:**

We propose a synthetic map with 11 linkage groups containing 489 markers (167 SSRs and 322 DArTs) covering 1197 cM. This first saturated map is proposed as a "reference *Musa *map" for further analyses. We also propose two complete parental maps with interpretations of structural rearrangements localized on the linkage groups. The structural heterozygosity in P. Lilin is hypothesized to result from a duplication likely accompanied by an inversion on another chromosome. This paper also illustrates a methodological approach, transferable to other species, to investigate the mapping of structural rearrangements and determine their consequences on marker segregation.

## Background

The banana (*Musa spp*.), including sweet and cooking bananas, is the number one tropical fruit, with a global production exceeding 100 million tons in 2006. It is also a staple food for more than 400 million people [1]. Largely due to technological requirements for transportation and agronomic performances, 45% of world consumption relies on a single genotype (cv. Cavendish), which is susceptible to the main *Musa *diseases [2]. It is therefore urgent to breed new, disease-resistant genotypes that can be cultivated with less pesticide.

Musa is a Monocot with four known genomes (A, B, S and T) and a relatively small genome size of 500-600 Mb in its haploid state. Two species, *M. acuminata *(2n = 2× = 22) and *M. balbisiana *genomes (2n = 2× = 22) participate to most edible triploid bananas and contain an A and B genome, respectively.

*Musa textilis *from Australimusa section (2n = 2× = 20) and *M. schizocarpa *(2n = 2× = 22), carrying T and S genomes respectively, are involved in few edible cultivars[3,4]. Cultivated triploid clones (AAA such as Cavendish, AAB such as plantain varieties, and ABB cultivars) are difficult to cross because of sterility, polyploidy, high heterozygosity, interspecificity and low gamete fertility, thus limiting banana improvement [3]. Sterility is generally associated to genome structural heterozygosity. These structural differences likely contribute to crossing barriers within the species. Consequently, the *Musa acuminata *complex has been divided into seven "translocation groups" [5].

The most widely distributed type is designated the "standard" or "central" group, because of its broad distribution in the *M. acuminata *species and in other *Musa *species [5]. In *M. acuminata, microcarpa subsp., banksii subsp*. and most *malaccensis *subsp. share this structure. The other six groups (Northern Malayan, Northern 1 and Northern 2, Malayan Highland, Javanese, East African) are defined on the basis of chromosome pairing during meiosis. Within each group, wild accessions share the same chromosome structure and are structural homozygotes, in contrast to most cultivated accessions. In previous characterizations, the inversions were not likened to "translocations", even if chromosome segment inversions was suspected [5]. The fertility of all cultivars is altered by their structural heterozygosity and sterility increases with the number of rearrangements/structural differences [5].

Despite the importance of a well established genetic map to sustain banana genetic improvement at diploid and triploid levels, this tool is presently lacking because of difficulties with *Musa *in developing a mapping population free of any structural rearrangement. The previous efforts [6,7] highlighted the likely presence of rearrangements but did not provide an interpretation in terms of the structure of the affected chromosomes.

The first mapping experiment with *Musa *produced a non-saturated genetic map [6], which exhibited 15 linkage groups with 77 markers, among which 36% significantly deviated from Mendelian segregation (p < 0.05). In that study, the F_1 _parent, selfed to generate the segregating progeny, was shown to be heterozygous for two reciprocal translocations. The second map was drawn from 89 individuals coming from a selfed *M. acuminata *diploid "M53". It displayed 11 linkage groups and also distorted markers [8]. The third map featured 14 linkage groups [7]; 59% of the 120 markers were skewed (p < 0.05) and the F_1 _hybrid used to generate the F_2 _population carried at least two translocations, if not three. Pseudo-linkages could have led to the establishment of oversized linkage groups comprising distorted markers supposed to be involved in the structural rearrangements [9].

A fourth map was to generate a refined *M. acuminata *parental map that could serve as a dense reference *Musa *genetic map containing the 11 expected linkage groups. Mapping was performed using a F1 population of diploid *Musa acuminata *genotypes. The female parent was the wild *M. acuminata *'Borneo', subsp. *microcarpa*, supposed to be a structural homozygous. The male parent was the cultivar *M. acuminata *'Pisang Lilin', subsp. *malaccensis*, exhibiting a Northern Malayan/Standard heterozygous chromosomic structure [5,10]. Therefore, the structural heterozygosity of the progeny, named Borli population, should be limited to a unique rearrangement. This work was enabled by combining methodological approaches (DArTs and SSRs) with analytical approaches (Neighbor joining trees) to determine the structure or large chromosomal rearrangements and their location in the genetic maps of the parents of the population.

## Results

### Meiotic configuration

Like many banana cultivars, the male parent *M. acuminata *'P. Lilin' contained structural chromosome rearrangements, while the wild female parent *M. acuminata *Borneo, is supposed to be free of any.

**Figure 1:**
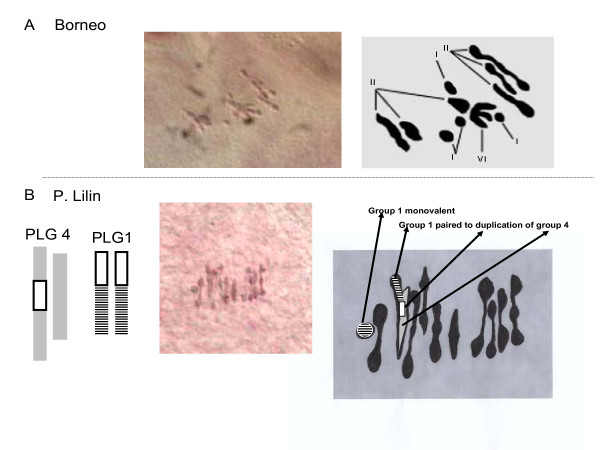
**Views of chromosome pairing during meiosis (metaphase I) and their diagrammatic illustration**. A - Borneo: the plate represents 4 monovalents (I), 6 bivalents (II) and one hexavalent (VI). The hexavalent is the result of association of three bivalents likely by their distal segments. B - P. Lilin: The plate shows 1 monovalent, 9 bivalents and 1 trivalent displaying a Y shape. This pattern suggests a connection point located in the proximal position.

Meiotic preparations of Borneo and P. Lilin were analyzed. They both displayed some Pollen Mother Cells (PMC) with normal chromosome pairing forming 11 bivalents (Table 1 configuration A) and some PMCs showing some degree of multivalent pairing. Borneo showed one cell displaying one trivalent and one tetravalent (configuration H), one cell displaying one pentavalent (Table 1 configuration G) and one cell displaying a hexavalent (Table 1 configuration F and Figure 1-A). On this basis, we infer that Borneo has at least two structural polymorphisms linking three pairs of chromosomes. This was not expected because Borneo, which is a seeded wild accession with good male and female fertility, was described as structurally homozygous [5].

**Table 1:** Meiotic configurations at metaphase-I and anaphase-I in the parents of the Borli population.

		Number of PMCs scored	
Metaphase-I configurations	Cell configuration	*M. acuminata *'Borneo'	*M. acuminata *'P. Lilin'
A	11 II	13	4
B	10 II + 2 I	1	3
C	9 II + 1 III + 1I	-	9
D	9 II + 1 IV (open)	1*	1**
E	8 II + 2 III	3	-
F	8 II + 1 VI	1	-
G	8 II + 1 I + 1 V	1	-
H	7 II + 1 I + 1 III + 1 IV	1	-
Anaphase-I configurations			
I	11/11 chromosomes	2	-
J	10/10 chromosomes + 1 bridge	-	1
Total no. of cells scored		21	17

I: monovalent - II: bivalent -- III: trivalent -- IV: tetravalent -- V: pentavalent -- VI: hexavalent.

*: IV open X shape; ** IV open Y shape. PMC: Pollen Mother Cells

Meiotic configurations of the P. Lilin parent revealed less complex features. The presence of various cells with only one trivalent (Table 1, configuration C, Figure 1-B) as well as one cell showing an open tetravalent (Table 1, configuration D) led us to tentatively assume one structural polymorphism in P. Lilin. In addition, the bridge observed in one PMC at anaphase-I (Table 1, configuration J) suggested the presence of one chromosome fragment inversion. It is noteworthy that no "closed tetravalent" was observed in these preparations. Therefore, it is not possible to establish the presence of a true translocation across chromosomes. The meiosis observations on P. Lilin are consistent with previous work [10,11] on this same clone that also drew the conclusion of an inversion.

### Marker polymorphism

#### SSR markers

The SSR marker polymorphism in parents was tested for the 395 primer pairs selected (357 from *M. acuminata *and *M. balbisiana *genomic SSRs, and 38 from ESTs). Two hundred and fifty-six primer pairs generated PCR amplicons, among which 181 had polymorphism detected, and exhibited clear and unambiguous single-locus amplification on the parents.

Of these 395 SSR markers tested, the 29 SSR markers defined on *M. acuminata *"Gobusik" have been extensively used in mapping [6,12] and diversity analysis [13-15]. The other ones are newly defined. This may explain that 76% of the former were mapped, while only 43% of the latter were usable.

Borneo was less heterozygous than P. Lilin. Of the 174 mapped SSR, Borneo displayed 126 heterozygous patterns (72%) as opposed to 151 for P. Lilin (87%); 103 of the segregating markers segregated in both parents (59%), while 23 SSR markers segregated only in Borneo and 48 SSR markers in P. Lilin. Genotyping data are available on GCP registry http://gcpcr.grinfo.net/index.php?app=datasets&inc=files_list.

#### DArT markers

The two parents and 92 progenies were hybridized on DArT array. Four hundred and eighty-five markers were found to be polymorphic out of the 11520 present on the array (4%). Among the 485 markers, 59 could be attributed to a linkage group but were impossible to map (inability to define phases, generation of negative distances and high value of marker mean square contribution ...). Among the 426 DArTs markers that were mapped, 144 (34%) were contributed by Borneo only, 228 (53%) were contributed by P. Lilin only, while 54 (13%) were contributed by both parents. In the reference map, 62 fully identical markers, probably resulting from redundancy, were discarded. Genotyping data are available on the GCP registry http://gcpcr.grinfo.net/index.php?app=datasets&inc=files_list.

### Anchorage between parental maps

**Figure 2:**
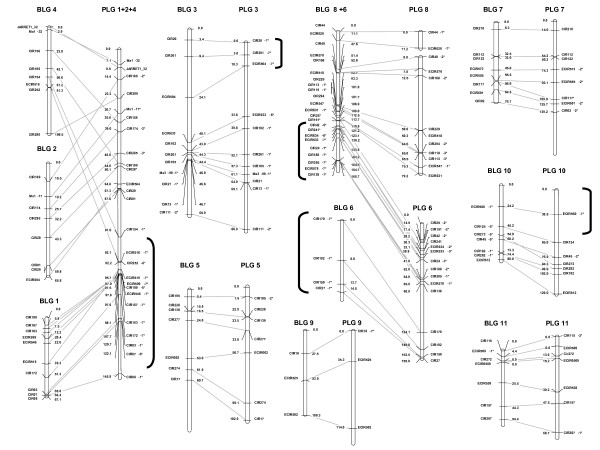
**Map of parents of the F_1 _Borli population**. Linkage group representation displays only anchor markers. Borneo linkage groups (BLG) were defined at LOD3.5 while P. lilin linkage groups (PLG) were defined at LOD 5. For Borneo molecular marker names are on the right side of each linkage group and genetic distances are on the left (cM Kosambi), whereas the marker names are on the left side and genetic distances on the right side of the linkages groups for P. lilin. Loci labeled with asterisks showed distorted segregation (1* P < 0.05, 2* P < 0.01, 3* P < 0.005, 4* P < 0.001, 5* P < 0.0005, 6* P < 0.0001, 7* P < 0.00005). Brackets] indicate segments with highly distorted markers (P < 0.0001). In SSR names, mMaCIR has been abbreviated to CIR, mMECIR to ECIR.

The attribution of markers to one of the two parents enabled development of two parental maps. As a first step towards a tentative synthetic map avoiding parent-specific pseudolinkages, the two parental maps were compared at different LOD scores using 133 common markers (79 SSRs and 54 DArTs markers) serving as anchors. The congruence between parental linkage groups was best at LOD 3.5 for Borneo and LOD 5 for P. Lilin (Figure 2). Five consensus linkage groups (i.e. LG 3, LG 5, LG 7, LG 9 and LG 11) were identified on the basis of the full co-linearity of the anchor markers. For the other groups, marker alignments or groupings differed between the parental maps. At LOD 5, the Borneo markers homologous to markers of PLG6 split into two groups. They still split at LOD 3.5, but some of them aggregated with markers homologous to those of PLG 8. The groups BLG1, BLG2 and BLG 4 in the Borneo map built at LOD 3.5 lump into a major group in the P. Lilin map (PLG1+2+4) even up to LOD 8. At LOD 9, the P. Lilin map exhibited 14 linkage groups and the grouping was no longer consistent with the Borneo representation.

The map based on Borneo female parent had 11 linkage groups that were delineated at LOD 3.5 with 261 markers (125 SSRs and 136 DArTs) (Additional file 1). The map spanned about 920 cM, with an average marker spacing of 3.8 cM. The largest linkage group comprised 59 markers whereas the smallest encompassed 9 markers. Of the 278 segregating markers initially tested, 8 DArTs remained ungrouped and mMaCir 120 was removed.

Regarding the P. Lilin parent, the map obtained at LOD 5 comprised 359 markers of the 379 initially tested (147 SSRs and 212 DArTs), distributed in 9 main linkage groups (PLG) (Additional file 2). The map spanned about 1081 cM with an average marker spacing of about 2.9 cM. The markers were not uniformly distributed, one major group (PLG 1+2+4) comprising 113 markers. Sixteen markers remained ungrouped, including 4 SSRs and 12 DArTs. Four more DArT markers (292027, 292284,292234 and 295644) were removed because they disrupted the order of the linkage groups (negative distances; suspect double recombinants).

### Segregation distortions

Twelve percent of the markers deviated from the expected Mendelian ratio (χ^2 ^test, significance p < 0.005) on the Borneo female parent (31/269), whereas this percentage reached 24% with the P. Lilin male parent (89/375). So, Borneo exhibited half the rate of highly distorted markers of P. Lilin. The distortions were of the same order of magnitude for SSR and DArT markers.

Skewed segregations affected different linkage group segments of the parental maps. For example, markers segregated without any distortion on BLG 1, BLG 2 and BLG 4, whereas half of the markers were highly distorted on PLG 1+2+4. Similarly, markers on the homologous Borneo group followed Mendelian ratio (1:1) while one segment on PLG 3 showed strongly skewed marker segregations (Cir20 to ECIR633, Figure 2). Conversely, Borneo was affected by highly skewed markers on the segmented BLG 6 while the corresponding loci on P. Lilin exhibited weaker distortions (Figure 2). Among the most highly significant distorted segments (i.e. p < 0.0005), allelic ratios of the markers varied from 1:2 to 1:5 depending on the linkage group and sometimes on the location within a linkage group.

### Linkage group tree representations

**Figure 3:**
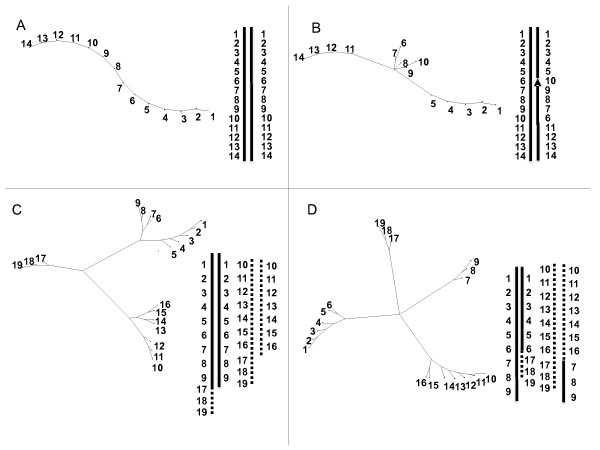
**Graphic representations of structural rearrangements on simulated data**. A: one structurally homozygous linkage group with 14 equidistant loci. B: as A but heterozygous for the inversion of segment 6 to 10 C: one non reciprocal translocation between two linkage groups: structural heterozygosity comes from translocation of segment 17 to 19 from linkage group 2 to linkage group 1 D: one reciprocal translocation of segment 7 to 9 from chromosome 1 to chromosome 2

**Figure 4:**
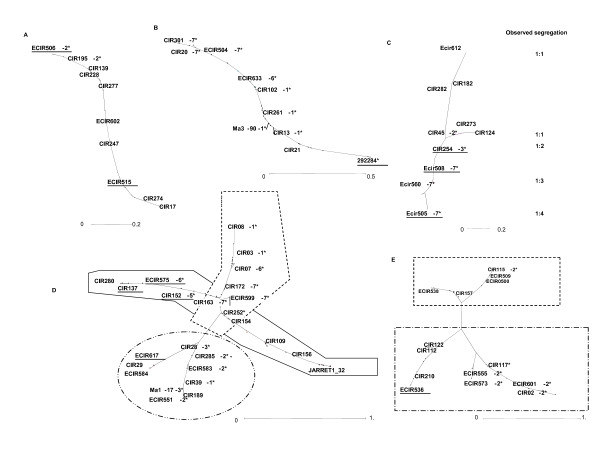
**Neighbor-joining trees designed from different linkage group data of P. Lilin**. A: PLG 5 showing perfect alignment of markers, B: PLG 3, C: PLG 10 showing inverted segment; D: PLG 1+2+4 showing anchor markers belonging to BLG 1 (- -), BLG 4 (--), BLG 2 (- · · -) on Borneo, E: example of artificial grouping of the independent PLG 7 (- · · -) and PLG 11 (--) at LOD 1. Markers on linkage groups are anchor markers, except underlined ones which are P. Lilin markers.

Adapted tree analyses provide an alternative representation of linkage groups. Trees have been drawn from simulated data of different features of chromosomal rearrangements (Figure 3 and methods). It was applied to all P. Lilin parental linkage groups defined by JoinMap^® ^4 at LOD 5. Figure 4 summarizes the different patterns obtained from the observed data. Most of the P. Lilin linkage groups displayed figures of homologous chromosomes (Figure 4-A) with alignment of markers along the NJ tree similar to that of Figure 3-A, even for PLG 3 (Figure 4-B) which displayed skewed segregations. Two atypical NJ trees were observed for PLG 10 (Figure 4-C) and PLG 1 + 2 + 4 (Figure 4-D).

**Figure 5:**
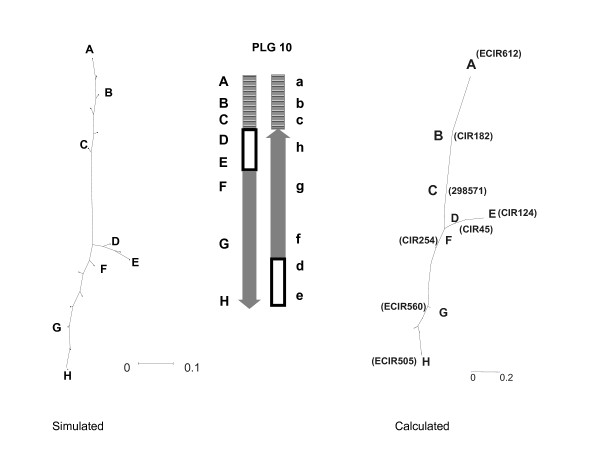
**Putative rearrangement event on PLG 10**. The figure presents the Neighbor-joining tree designed from Kosambi distance calculation, the putative scheme of the rearrangement, and the simulated Neighbor-joining tree obtained with this kind of rearrangement. In SSR names, mMaCIR has been abbreviated to CIR, mMECIR to ECIR.

Concerning PLG 10, we propose that a nested inversion pattern differentiates the two homologous chromosomes (Figure 5). This inversion should fit the observed cytogenetical inversion features. When this possibility was subjected to NJ tree analysis, we observed a good homology between the observed and simulated trees (Figure 5).

The representation of PLG 1+2+4 (Figure 4-D) is more complex. The homologs of the anchor markers from BLG 1 and BLG 4 are tightly linked, while the homolog markers of BLG 2 are loosely linked to those of BLG 1 and BLG 4. Actually, the aggregation of PLG 2 (Figure 4-D) looked very similar to those of independent groups artificially grouped at LOD 1(Figure 4-E), probably indicating a pseudo-linkage due to skewed markers [16], and PLG 2 is independent from PLG 1 and PLG 4.

**Figure 6:**
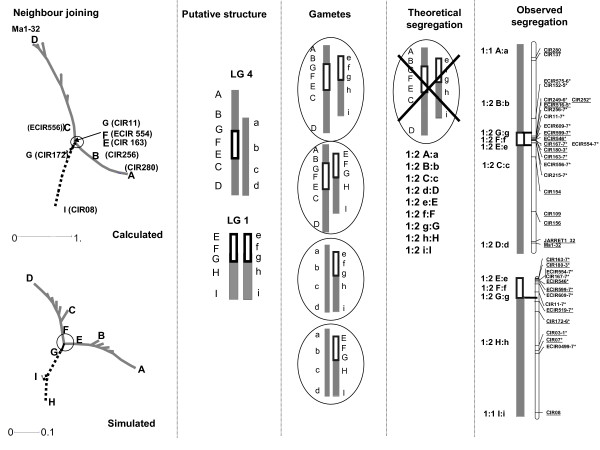
**Putative rearrangement event between PLG 4 and PLG 1 in P. Lilin**. The observed segregations are given along the linkage groups. Loci labeled with asterisks showed distorted segregation (1* P < 0.05, 2* P < 0.01, 3* P < 0.005, 4* P < 0.001, 5* P < 0.0005, 6* P < 0.0001, 7* P < 0.00005). Main gametes do not take into account the different allelic combination generated by recombination between homologous segments. Underlined loci are anchor markers. The solid grey line corresponds to PLG 4 and the dotted lines to PLG 1. In SSR names, mMaCIR has been abbreviated to CIR, and mMECIR to ECIR.

In contrast, the pattern of markers from PLG 1 and PLG 4 suggests a "translocation" of markers from PLG 1 into PLG 4 in proximal location. The rearrangement is non-reciprocal as the NJ tree (Figure 4-D) would display three arms instead of four in case of reciprocity as observed in *Prunus *[17] and derived from a simulation as shown in Figure 3-D. Furthermore it looks like the typical Y image of Figure 3-C. The best hypothesis would be the existence of a duplication, as suggested by Wilson [11], of a segment of PLG 1 into PLG 4 (Figure 6), but not a true translocation [10] as we neither observed the segregation ratio nor the genotyping profiles expected with a translocation. This hypothesis of segment duplication, associated with lethality of the type of gamete containing the duplicated segment in heterozygous configuration (Figure 6) is also consistent with the observed allelic pattern and allelic frequencies and is in agreement with meiosis configurations (Figure 1-B). The expected Mendelian segregation (i.e. 1:1) observed at the ends of PLG 4 and PLG 1 might result from higher recombination rates in telomeric segments [18] associated with the progressively decreasing rearrangement effect on segregation ratios with increasing distance from the inserted segment. Furthermore, the genetic distance observed between markers within this duplicated segment is low, as found in other studies [19]; [20]. Taking into account this low recombination frequency in the duplicated segment and the increasing frequencies of recombination from the inserted segment to the chromosome ends, the theoretical and observed NJ trees (Figure 6) are very similar.

On PLG 3, we observed highly skewed markers (P < 0.0005) especially at the end of the linkage group, while corresponding markers on the same Borneo group are not distorted (Figure 2). These distortions are located in segments that are collinear when mapping with JoinMap^® ^4 and that also align along the NJ tree representation (Figure 4-B). Therefore we propose as a hypothesis that this region may be subjected to gene selection, meiotic drive or epigenetic effect rather than affected by structural rearrangements.

**Figure 7:**
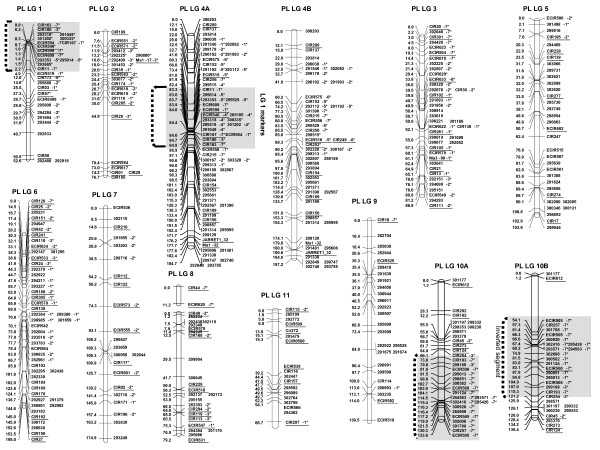
**Putative P. Lilin map built at LOD 5**. Molecular marker names are on the right side of each linkage group whereas genetic distances are on the left (cM; Kosambi mapping function). Loci labeled with asterisks showed distorted segregation (1* P < 0.05, 2* P < 0.01, 3* P < 0.005, 4* P < 0.001, 5* P < 0.0005, 6* P < 0.0001, 7* P < 0.00005). Anchor markers are underlined. The 4A and 4B linkage groups represent both chromosomes of the structural heterozygous pair of the chromosome 4. The linkage groups 10A and 10 B represent the both chromosomes of the structural heterozygous pair of the chromosome 10. In SSR names, mMaCIR has been abbreviated to CIR, and mMECIR to ECIR.

Overall, based on NJ tree analysis, allele segregations and cytogenetical studies, we propose the presence of two structural rearrangement events for P. Lilin. The first is assumed to be a segment duplication of PLG 1 into PLG 4 (Figure 4-D and Figure 6), instead of a translocation. The second can be a translocation of a PLG 10 segment into itself (Figure 5). Figure 7 presents a putative map of P. Lilin integrating these structural rearrangements.

For Borneo, at LOD 3.5, representations given by the NJ tree seem to indicate a translocated segment from BLG 6 to BLG 8 (Additional file 3). To reproduce the pentavalent or hexavalent pairing features (specific to rearrangement events involving 3 chromosomes) observed on meiosis plates (Figure 1-A), we need to decrease the grouping LOD score down to 2.9. In this case, parts of BLG 6, BLG 7 and BLG 8 are associated. Nevertheless, even in this case, we did not find any chromosome rearrangement model that could explain the very high distortions (P < 0.0005) observed on homolog markers of PLG 6, while BLG 7 and BLG 8 did not display any. Furthermore, all homologous markers of PLG 6 only aggregate at LOD 1. The third group indicated by cytogenetical studies is still not clearly found, nor is the kind of rearrangement that can lead to such a feature.

### Synthetic map

**Figure 8:**
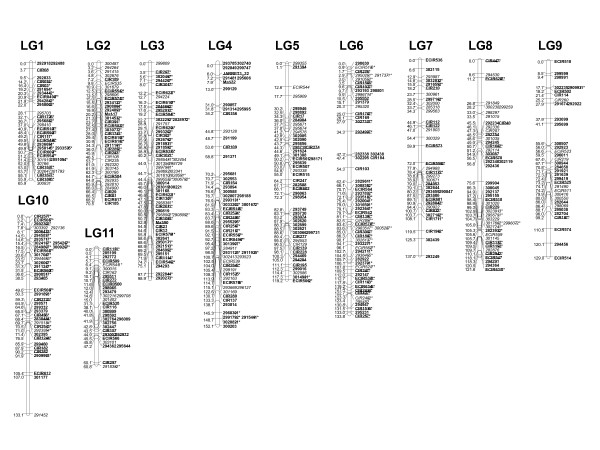
**Reference *Musa *map built from *M. acuminata *P. Lilin and *M. acuminata *Borneo maps**. The map has been built using JoinMap^® ^4 software, Kosambi distance calculation. Molecular marker names are on the right side of each linkage group whereas genetic distances are on the left (cM, Kosambi mapping function). Anchor markers are in bold and underlined, markers polymorphic only on Borneo are in italic; those polymorphic only on P. Lilin are in bold. In SSR names, mMaCIR has been abbreviated to CIR, and mMECIR to ECIR.

A final synthetic map was constructed at LOD 5 (Figure 8). It was first established from the aligned parental linkage groups described above (i.e. LG 3, LG 5, LG 7, LG 9 and LG 11). For the remaining linkage groups, the grouping and the marker alignments kept as skeleton were chosen from the parent assumed to be free from any structural rearrangement on the considered linkage group. The absence or presence of putative structural rearrangements was assessed with NJ tree representation and segregation analysis. Priority was given to linkage groups exhibiting a linear NJ tree and Mendelian segregations. A few cases of linear NJ tree with limited distorted segregation were also retained.

From the 426 initial DArT markers, 62 fully identical markers were discarded and 8 DArT markers remained ungrouped. Five SSR markers and 34 DArTs associated with structural rearrangements in one parent were discarded from this reference map because they disrupted its construction (i.e. negative distances, suspect double recombinants, high mean square contribution...). Therefore, some markers present in the parental maps are absent from the reference map. The observed recombination frequency between two markers, one located in the structural rearrangement and one outside, aggregates the results of two different situations: one is linkage, the other one is independency. In practice, the recombination frequency is the mean of recombination of linked markers (REC < 0.4) and of independent markers (≥0.4) in the reference map. Consequently, these markers disrupt both the calculation distances between markers and the ordering when compared to data obtained from the non-rearranged parent.

Altogether, this synthetic map includes 489 markers (167 SSRs, 322 DArTs) among which 132 are anchor markers. It is divided into 11 linkage groups covering 1197 cM. The markers are distributed with a mean of 38 markers per linkage group and an average marker spacing of 2.8 cM.

## Discussion

This study reports an important effort in marker development and linkage analysis. SSRs provide co-dominant, multi-allelic, locus-specific markers which simplify both the construction of each parental genetic map and the comparison between the two parental maps. DArT provide dominant markers which are generally well-distributed in the genome and very cost-effective [21,22]. They efficiently contribute to map saturation and they constitute an asset that can easily be used for other materials in the future. The main difficulty of our study was the general occurrence of segregation distortions and the risk of pseudolinkages.

Distortions from expected Mendelian segregation have been observed in both inter-specific and intra-specific derivatives with different magnitudes. They can have multiple origins, including structural rearrangements [6,17,19,23-29].

In the genus *Lens*, for example, distorted markers were observed in different linkage groups, but only one translocation was detected by cytogenetical studies and pollen viability analysis; its location was defined on the basis of marker locations in different crosses [23]. In *Helianthus*, [26], reciprocal translocations were described by conjugating observations of abnormal pairing in meiosis, studies on pollen viability and mapping data. The latter revealed an abnormally large linkage group covering close to half of the map. Nevertheless, the causes of the observed segregation distortions often remained unclear. In *Prunus *inter-specific crosses skewed markers were located on a reciprocal translocation [17]. The hypothesis was validated by studying pollen fertility in the segregating progeny and by cytogenetical observations during meiosis. Most examples show that an array of methods is generally needed to differentiate between the different causes of segregation distortion.

In *Musa*, earlier studies did not determine the causes of segregation distortion [6,12]. Our use of NJ tree representations helped sort between the segregation distortions linked to structural rearrangements and those due to other phenomena such as gene selection, meiotic drive or epigenetic transmission effects. This use led to identification of one likely case of local direct selection on PLG3 and a couple of likely structural rearrangements.

Earlier cytogenetic studies of meiosis in P. Lilin [10,30] described this cultivar as a structural heterozygote featuring one translocation and at least one inversion on the basis of trivalents and bridges. In subsequent observations, Dodds and Simmonds [30] and Shepherd [5] suggested that one of the exchanged segments contains a small sub-terminal inversion, but Wilson [11] stressed that no closed tetravalent was observed and suggested a duplication rather than a translocation. Our interpretation features a duplication between PLG 1 and PLG 4 and an inversion within PLG 10; it is therefore fully in line with earlier inferences.

The structural status of Borneo is less clear. Early cytogenetical studies described Borneo as a structural homozygote [5], in consistency with its full fertility; yet some rearrangements are suspected as well as the presence of a unit with "one of both arms rather short" [5] Our cytogenetic observations suggested that it is heterozygous for at least two rearrangements involving three linkage groups. Some BAC-FISH experiments should be undertaken, as was done for *M. acuminata *[31], among the parents of an F_2 _mapping population 'Calcutta 4' and 'Madang' [7] in order to physically assess the nature of the rearrangements. Based on our results, the investigations should focus first some of the linkage groups highlighted above: PLG1, PLG4, BLG 6, BLG 7 and BLG 8. This is possible with the availability of several BAC libraries [32,33].

In our effort to produce a saturated map of *Musa*, we initially meant to study a progeny involving one structurally homozygous parent. The unexpected structural heterozygosity of Borneo induced additional complexity; yet it is likely that the rearrangement patterns involved different chromosomes and did not overlap in the two parents. Thus, we think our synthetic map can be a valuable reference for a *M. acuminata *genetic map. Whether this map corresponds to a "standard structure" [5] representative of the ancestral state requires confirmation with other mapping studies involving other germplasm compartments. The finding of structural heterozygosity in the "wild" Borneo challenges the idea that the wild forms have developed several genome arrangements in distinct populations, identified as subspecies, which then cause sterility and favor domestication among hybrids. Borneo belongs to the Microcarpa subspecies and has been found recently [34] to include some Bansksii subsp. alleles, indicating probably an intersubspecific origin. The structural heterozygosity in this case should be sufficiently limited to not affect fertility.

It may be difficult to establish the ancestral configuration by comparing only A genome forms; the use of a B genome representative as an outgroup will certainly be helpful.

The case of the relation between linkage groups 1 and 4 in P. Lilin sheds light on particular aspects of structural polymorphisms in banana. Our current interpretation is the duplication of a segment featuring at least fifteen markers, including eight SSR loci, even if the breakpoint on PLG1 should be refined more precisely. Coding the markers/alleles as co-dominant (forcing a single locus) or as dominant (allowing two loci) in the segregation data does not modify the global size of the segment: these markers span on about 1 cM in P. Lilin. In contrast, they span on about 15 cM in Borneo.

If the cause for the distortions and the pseudolinkages is indeed this duplication, it implies that P. Lilin has three copies of the loci involved in the duplication. Yet no case was found where one of these loci displayed three distinct alleles, meaning that the duplication features a haplotype that is conserved along a segment which spans 15 cM in the Borneo configuration. This would mean that this duplication is recent enough for bearing no trace of SSR mutation nor of recombination with other haplotypes. P. Lilin is clearly incompletely fertile, but it was sufficiently fertile to produce the large number of progeny that we analyzed. We cannot exclude that this configuration is unique to P. Lilin, neither can we exclude it from a wider group of wild accessions that exchange genetic material. This leads to the question of the population dynamics of structural heterozygosity: if a heterozygous configuration leads to genetic map constrictions, and if it does not hamper reproduction, a fraction of the progeny that is again heterozygous should display the same genetic constriction. This could induce specific linkage disequilibrium in the area of rearrangement. Therefore, it can be worth testing whether this type of evidence, namely linkage disequilibrium among stretches of markers, can help infer the distribution of structural rearrangements in the whole species.

As a more direct route, strong segregation distortions induced by structural rearrangements can also be monitored using small-sized progenies (30-50 individuals) and be subjected to the neighbor joining approach that we have used.

## Conclusions

We present here the first dense genetic map of *M. acuminata *with the expected eleven linkage groups, on the basis of a synthesis between two parental maps featuring distinct patterns of segregation distortions. This map can serve as a tentative reference for further studies. It displays 167 SSR and 322 DArT loci, covering 1197 cM with an average density of one marker for 2.8 cM.

It will be central in further analysis of the genome of *Musa*. Current projects plan a complete sequencing of the A genome of a particular doubled haploid derived from Pahang, a Malaccensis genotype. In that project, density of the genetic map will be further increased.

This synthetic map is also accompanied with hypotheses for structural rearrangements and selection pressures which occur in the two parents, with more precision in P. Lilin. Our understanding of these rearrangements is not complete, but our findings do provide testable hypotheses for molecular cytogenetic studies for visualizing structural polymorphisms. It is clear that more genome structure analyses are needed for understanding the patterns in the A genome, which are very important for breeding activities. The SSR markers provide a framework for selecting polymorphic markers for new mapping studies, whereas the DArT markers will efficiently complement a loose SSR selection for efficiently filling potential gaps. Therefore, new maps can be quickly generated when new progenies are available. A complementary *ad hoc *effort could also be needed to develop co-dominant markers which can reveal dosage effect in order to help resolve patterns which involve structural rearrangements.

## Methods

### Material

The segregating population, named "Borli" in this paper, was obtained from the intraspecific cross of two heterozygous diploid accessions [*M. acuminata *'Borneo' x *M. acuminata *cv. 'Pisang Lilin']. The parents were chosen based on a combination of different criteria such as genetic distance (0.8 from simple matching distance based on 20 SSR markers, data not shown), allelic heterozygosity (>60% for each parent on the basis of 20 SSR study, data not shown) and putative structural heterozygosity. The *M. acuminata '*Borneo' female parent belongs to *subsp. microcarpa *and is a "wild" seeded accession originated from Indonesia. It was shown as a structural homozygote belonging to the "standard group" [5]. The *M. acuminata '*P. Lilin' variety is a male fertile *M. acuminata subsp. malaccensis *derivative originating from Malaysia, and supposedly heterozygous for one translocation as a hybrid from Central and North Malayan translocation groups [5,10].

The cross was made in Guadeloupe (French West Indies). The F_1 _hybrids were germinated *in vitro *[35] and the 268 plantlets then transferred to a greenhouse in Montpellier. The ploidy level of all individuals was assessed by flow cytometry and chromosome counts; one case of triploidy was observed and the corresponding individual was removed. One hundred and eighty randomly chosen individuals were genotyped for segregation analysis.

### DNA isolation

Leaves were harvested for DNA extraction 2 months after plant transfer to the greenhouse. 3 g of frozen material was ground with liquid nitrogen with a mortar and pestle following the modified Matab method [36]. The DNA was re-suspended in PCR grade water after isopropanol evaporation.

### Ploidy level

As polyploid or aneuploid individuals are often observed in *Musa *progenies [37,38], the ploidy level of the whole progeny (i.e. 268 plants) was determined by flow cytometry. Nucleus extraction and staining were prepared with a CyStain UV ploidy kit (Partec CyStain UV ploidy kit, Partec GmbH, Münster, Germany). The analyses were performed with a PAS flow cytometer (Partec) equipped with an HBO lamp for UV excitation. A leaf sample of each individual plant was chopped together with leaf tissue of *M. acuminata *cv. Cavendish clone (2N = 3× = 33) as an internal reference. Each plant underwent two independent measurements.

As a cross reference, chromosome counts on root tip squashes [39] were also performed on a sample of 20 individuals.

### Cytogenetic analysis

Immature anthers were excised from male flower buds of both Borneo and P. Lilin clones from 28 to 33 days before anthesis, and fixed overnight in a 1:3 acetic acid: ethanol solution saturated with ferric acetate. They were then transferred into 70% ethanol and stored at 8°C in this solution.

Meiotic chromosomes were studied in pollen mother cells (PMCs) squashed in aceto-carmine staining solution. Observations were performed on metaphases I containing enough well flattened cells to complete bright-field analysis at ×1000 magnification or differential interferential contrast analysis. An average of 15 PMCs per clone were scored for chromosome rearrangements.

### SSR markers

We tested 38 SSR markers from a SSR enriched library constructed from *M. acuminata *'Gobusik' (mMaCir01 to mMaCir 45 [40], Jarret 03 to Jarret 1_32 and Ma1_17 to Ma 3_90 [41] and 205 SSR markers from another SSR enriched library for (CA)n and (CT)n microsatellite and constructed from *M. acuminata *'Calcutta 4' and *M. balbisiana *'Pisang Klutuk Wulung' (mMaCir101-EMBL AM950326 to mMaCir307 -EMBL AM950533). The SSR markers with their primer pairs are presented in Table 2. We also used 143 markers from a *M. acuminata *'Calcutta 4' EST library (mMECir501-EMBL FM878660 to mMeCir642-EMBL878794). The other SSR markers came from other EST sequences (mMECIR491 to mMECir500; Table 2 and [42]) from *M. acuminata *"Cavendish".

**Table 2:** Characteristics of the mapped SSR loci from a *Musa *genomic library (mMaCIR102 to mMaCIR 305) and from EST sequences (mMECIR0494 to mMECIR0500).

SSR	EMBL	motif	LG	forward primer	reverse primer	Ann (°C)	Size (bp)
mMaCIR102	AM950328	(AG)10,(TG)5	6	TGTTGGATTGGCTTCATC	CTTCGTTCAATGGTCTCCT	55	220
mMaCIR103	AM950329	(CT)14,	3	CCTCTTCTCCCTGTGTTG	CGGTTTAACATACCTATTCTTG	54	179
mMaCIR105	AM950331	(CA)8,(CT)15	6	CATCCACTTGCTTTTCCA	CTTCACGGCTTCCACA	56	264
mMaCIR108	AM950334	(CA)7,(CA)4,	2	ACGCATGGTAAAGTGGAA	ACATTCAAATCACGTTGCT	55	111
mMaCIR109	AM950335	(CA)13,	4	ACTCTAGTTCCAGAATAACTCCA	CAATCTTCATTAGCCAGTTGT	55	204
mMaCIR110	AM950336	(AC)7,(GA)6,	3	GGTGAACTGATGTGCGA	TCTTTCAACGGAATAAGCA	55	244
mMaCIR111	AM950337	(CA)8,	6	TCGTATGGAACAACAGTCC	CTTTCACCTTCAAACAGCA	55	137
mMaCIR112	AM950338	(CA)5,(CA)15	7	GTTCGGCTGGAGGTAGTT	AAGAACACGAAGGCAGG	55	330
mMaCIR113	AM950339	(CA)10,	10	TCAAGTATTTCACCGTATTGC	TTACCACCCTGTCATCTTTC	55	207
mMaCIR114	AM950340	(AC)7,(CT)28	8	GCAAGCCAAAGGGAA	ACCAACAAAGAATGGTGTAA	54	222
mMaCIR115	AM950341	(CA)2,	11	CAAGAGACTACCACCGAAGA	TGATTCTCACGACGTATGG	55	114
mMaCIR116	AM950342	(TC)2,(TC)20	11	ACACAAAGAAACCAGCCA	CGTCCCATCGTCTCCT	55	202
mMaCIR117	AM950343	(TC)20,	7	GTTTGTGGAATAAGTGGGAA	ATGAGGGAGTTAGTGGTGG	55	214
mMaCIR119	AM950345	(CA)9,(TA)6,(CA)5,	10	TGAAAAGCAATCCAACCT	ACCCTGAAATGTTTGTCTTT	54	395
mMaCIR122	AM950348	(GT)8,	7	CGGTGACACTGGAAGGT	CAACTGAAGAACTGCCACTAA	56	204
mMaCIR124	AM950350	(AC)7,	9	ACCTTGACAGCCCTCTTC	ATCAATCATTTCTGGGGTT	55	63
mMaCIR129	AM950355	(CA)6,	5	CTAACCTTTGATTCTGTTTG	GTCCCTGATACACCATTC	50	214
mMaCIR130	AM950356	(TG)17,	6	TTAAACGTCTCCGTGTCTTC	TTGCATGAGGCTGGG	56	311
mMaCIR137	AM950363	(TC)12,	4	CGTATTCTACATCTGCTTCTTT	GCAGTGATTAGGTGATGATTT	54	223
mMaCIR138	AM950364	(CA)7,	3	TCATTCTCATGCGGAACT	CGGTGGATGTTGTTGG	55	173
mMaCIR139	AM950365	(GA)18,	5	TCGTCCCCTACTGCCT	ATGCTTCCGTTTGGCT	55	187
mMaCIR150	AM950440	(CA)10	3	ATGCTGTCATTGCCTTGT	GAATGCTGATACCTCTTTGG	55	238
mMaCIR151	AM950441	(CT)21	3	TATCCACCTCCTGGCAC	GCCAAACATCACCCAAC	55	172
mMaCIR152	AM950442	(CTT)18,(CT)17,(CA)6	4	CCACCTTTGAGTTCTCTCC	TTTCCCTCTTCGATTCTGT	55	163
mMaCIR154	AM950444	(CT)17	4	CATTCAGCATGGAAACCT	CTTCCTCAAACTGCTCCTC	55	311
mMaCIR156	AM950446	(TG)23	4	CTTTCTGAAGGAAATTCTGAC	AGTGCAGCCCAATGAA	54	210
mMaCIR157	AM950447	(CA)9,(TA)7	11	TGGTATTATTTCATAGCCCTTC	ATGGTATTGTTGGATGGTGT	55	272
mMaCIR162	AM950452	(CA)8	11	CTGCCTGTCCCACGA	GCGGCCATCATAATTCC	57	161
mMaCIR163	AM950453	(AC)14	1	TGAAACAATCTTCATCAGCT	TCTGGACTTGGATGCTATTT	55	247
mMaCIR167	AM950367	(AC)7,	1	CACTTCCACCTCTGCATC	GGTCTACTAACTTGAACACGAAC	55	336
mMaCIR168	AM950368	(CA)7,	10	GCACCAAACCAGTCCTAC	CGTCTCAGTTGCCGTG	55	243
mMaCIR169	AM950369	(CT)14,(CA)1	3	TTTGGAGGAGACCATGATT	GCATTACATATCCTGCCTTT	55	297
mMaCIR170	AM950370	(CA)8,	3	GGGCCTCCATAAGCAA	ACTTACCTTCCTGCCCAC	55	202
mMaCIR171	AM950371	(CA)5,(GA)10	7	GTAATACAAGTCTTCAGAGCAT	CTGTTTCGCCACTATCTT	51	192
mMaCIR172	AM950372	(CT)19,	1	CAGCTAATGCCAAACCC	CGACTTCGAGCGAGC	55	258
mMaCIR174	AM950374	(AG)13,	2	GAACCCACCTCCCTCTT	TGGGATTCCTGAGTGCT	55	167
mMaCIR180	AM950380	(CA)7,	1	GCCTCAGCCTCATCATC	CACCCACTCGACCCA	55	226
mMaCIR182	AM950382	(TC)22,	9	AACGCTTCTGCCTTGTT	TGAGACGTATTGCCCTAGTT	55	150
mMaCIR184	AM950384	(TG)7,	3	TGTCATCGGCATAGACTG	TGGAATTGAACTGAAGCC	54	314
mMaCIR185	AM950385	(TG)8,	2	CATTTCTATTCCCAGTCCC	CCAATGTTACTTCCATGCT	54	181
mMaCIR188	AM950388	(TG)9,(TA)7,	3	GTGCTTGTTCGCTTGTTT	AGCCCAAGTATCCCACC	56	160
mMaCIR189	AM950389	(CT)3,(CT)16	2	GGGAGGGCAGAGGAA	GCCGAACTTGGTAATGTG	55	259
mMaCIR192	AM950458	(TG)8	3	TGACCTAGCACAACGCA	GCTTATGTTTCATCGCCTT	56	133
mMaCIR193	AM950459	(AC)8	9	TGTCCCTATCTGTCCTCTTT	CGCTTTGGAGTGTGCT	54	301
mMaCIR195	AM950461	(GA)11,(GA)6	5	GAATCGCCTTAGTCTCACC	TCATGTGCTCCCATCTTT	55	285
mMaCIR196	AM950462	(TA)4,(TC)17,(TC)3	7	GCTCCAAACCTCCCTTT	CGATGCCACACTGGAC	55	173
mMaCIR210	AM950476	(GA)3,(TG)12,(AG)5	7	GGAAGGTGGCATGAAAG	TAACCTGATACCCATGTATTGA	55	319
mMaCIR214	AM950480	(AC)7	10	CCATTGAGAGATCAACCC	CTATTTGACGTTGGTGGTC	54	107
mMaCIR215	AM950481	(GT)7,(AT)3	4	AAGTTGGAGATATAGAATGGGT	TCCAGTGAATATGGATCAGT	54	327
mMaCIR219	AM950485	(GA)18,(AC)1	8	GGGTAAGCTCAAGATGGAA	CAGACGCTAAACGACACC	55	320
mMaCIR228	AM950494	(CT)18,(AC)7	5	CAAGCATGTTAGTTTGGGA	AAGGTGCATCCAAGGG	55	197
mMaCIR229	AM950495	(GA)22	10	CTGGGTTCCTCACCTTCT	GAAACACCATGTCCCAAA	55	253
mMaCIR235	AM950501	(CT)7,(CT)8	2	CCATCCCAGGCCATA	GCCCAGAGTCCGAAAG	55	329
mMaCIR241	AM950503	(TC)20	3	GCTAAGCATCAAGTAGCCC	ACGAACAAGCAATCAAAGTAG	55	297
mMaCIR247	AM950395	(GT)10,	5	AATGGATTGGGCATCAG	GGAGGGAGGAGGGTTT	55	178
mMaCIR248	AM950509	(TG)6,(GA)6,(AG)8	3	ATGCCTGCTACCACCTC	GCAGTTCCACAGTCCAAG	55	251
mMaCIR249	AM950396	(TG)9,	4	TGTATTGTATCCCTAATGTCCC	CCTTACTAGCCAATTACGTGAG	56	279
mMaCIR252	AM950510	(TC)9,(TC)3	4	TCGTAAGCGAAAGGTCG	CGAACGCACTACCACTATG	56	180
mMaCIR254	AM950512	(CT)25	9	CATGGAGGGTTAGGAGC	ATGCTTATTCTATGGTGGTTG	54	180
mMaCIR256	AM950399	(CA)7,	4	TTGCGGGAAACTGCT	GTTGCACTGCCCACTT	54	280
mMaCIR257	AM950400	(CA)7,	9	CTTTACCGAGTTGAGGG	TCATATCAGAAGATAGCCAA	51	234
mMaCIR260	AM950515	(TG)8	8	GATGTTTGGGCTGTTTCTT	AAGCAGGTCAGATTGTTCC	55	189
mMaCIR261	AM950516	(CA)13	6	TATCAGGCATACGTTCTGTAG	AAAGAAGGTGGGTGATAGG	54	202
mMaCIR264	AM950519	(CT)17	4	AGGAGTGGGAGCCTATTT	CTCCTCGGTCAGTCCTC	54	235
mMaCIR272	AM950408	(AC)6,(CT)5,	11	CTCACCGGATGGCAC	GGCATTAAGTTTCAGGAATAAG	55	171
mMaCIR273	AM950521	(TC)22,(CT)6	9	TGGTTGAAGATTCCCAT	GATCAAGAGGTGACAAACC	53	211
mMaCIR274	AM950409	(AC)11,	5	TAGCTCTTTCAACACTCTCATC	CTGGAGGCAGCGAAC	54	150
mMaCIR277	AM950523	(TG)12	5	ACGATAGGATTATTGGCTGT	GGCTCTTAATTTGACAAGAA	54	212
mMaCIR280	AM950412	(TC)7,(AC)7	4	GGGTCCCTGTTGGCT	TTGCAGATTAGGGTGGG	55	221
mMaCIR282	AM950414	(AG)8,(AG)3,(TG)	9	CATCCTGTTGCTCCCTC	AAGAATCTAGCAGCATCCAA	55	209
mMaCIR285	AM950416	(TC)21,(AC)7,(AC)5	2	ATTGCCATGATTGACCC	TACGGCTCCTATCGTCC	55	183
mMaCIR287	AM950526	(TG)7	10	TTTAAGAATCCCTCGCTTT	ACAGATGACGAACAAACTACC	54	203
mMaCIR289	AM950418	(GT)8,(TC)3,	3	TTGCTTCCTGTAACATCTCC	GGTCTGGGTGAAGGCA	56	205
mMaCIR294	AM950421	(AC)7,(TC)8,(CA)5,	10	CACGAGTCATAATCCAGTCA	GTTCAAAGCTCGTTGGG	55	178
mMaCIR297	AM950424	(TC)9,(AC)13,(CA)9	11	GAACTCGGATTGTTCCTTT	AGGCTGATGGTAGCGAG	55	173
mMaCIR301	AM950427	(TG)11,	6	CATGATGTTTGAGTTTGC	CTGGAAAGCAACACCG	54	166
mMaCIR305	AM950429	(CA)5,(TC)6,(GA)3,	3	CCGATCAATTCAGCCA	TATGAGCAAGAACAGCCC	55	299
mMECIR0494	pCav22	(TG)6c(GT)12	9	CCATGATACGGGCTTACGA	TCAATTACCAGCATCCTTACTT	56	266
mMECIR0496	ATPVScl3	(TA)7	4	CGCCACATAAGGCTCCCT	GTCGCCATCTCCTTGAA	55	176
mMECIR0498	SSHBSVban9a08	(GT)8nnnnn(TTC)13	6	CGGGGTCGTGTCTTAGGAA	GCAATCACACGGATACCTC	56	183
mMECIR0499	SSHBSVban9a08	(AG)8)	1	CGCTTGCCTTTGGTTGTG	CCAGTAGACGCCAATGC	56	172
mMECIR0500	SSH didier cl3	(AG)9	11	CCAGCAGACGCACACAAA	GCAACTGCAAATGAGGG	56	150

Calculated primer annealing temperature (Tm) and expected product size in reference variety (i.e. *M. acuminata *'Calcutta 4 and *M. balbisiana *"Pisang Klutuk Wulung" for mMaCIR102 to mMaCIR305 and *M. acuminata *'Cavendish' for mMECIR0494 to mMECIR0500). Expected size does not take into account the M13 tail (19 bp) added to each 5'end of forward primer. LG: linkage group; Ann: Annealing temperature used.

For a given SSR locus, the forward primer was designed with a 5'-end M13 extension (5'-CACGACGTTGTAAAACGAC-3') in order to generate fluorescent amplicons after fluorescent dye hybridization. Primer pair tests were performed on a 96 well PTC 100 thermocycler using the same protocol and reaction mixture as described below, in a final volume of 20 μl.

The PCR amplification was performed in a 384 well Eppendorf mastercycler with 10 ng of *Musa *DNA in a 10 μl final volume of buffer (10 mM Tris-HCl (pH 8), 100 mM KCl, 0.05% w/v gelatin, and 2.0 mM MgCl2) containing 0.08 μM of the M13-labelled primer, 0.1 μM of the other primer, 160 μM of dNTP, 1 U of Taq DNA polymerase (Life Technologies, U.S.A.) and 0.06 μM of M13 primer-fluorescent dye IR700 or IR800 (Biolegio, Netherlands).

The SSRs were amplified by couples. Two SSR markers were amplified at the same time in a 384 well plate under touchdown PCR conditions: initial denaturation at 94°C for 60 s; touchdown cycles were performed at a rate of -1°C/cycle. The first cycle was conducted at the highest primer melting temperature (TM), and the last cycle of the touchdown program was conducted at the lowest primer pair TM. These initial cycles were followed by 35 cycles at 94°C for 30 s, [lowest TM -1°C] for 60 s, and 72°C for 120 s; and a final elongation step at 72°C for 5 min. For example, if the TM of the first pair was 58°C and the TM of the second pair was 53°C, we performed the first cycle at 59°C; the 7 following cycles were performed in touchdown at 53°C. PCR was achieved with 35 more cycles at 52°C.

IR700 or IR800-labeled PCR products were diluted 8-fold and 5-fold respectively, subjected to electrophoresis in a 6.5% polyacrylamide gel and then sized by the IR fluorescence scanning system of the sequencer. For each run, a ladder (range 71 to 367 bp) was added at the edges of the gel. The gel pictures were analyzed using AFLP-quantar Pro software [43] with two independent readings.

### DArT markers

DArT arrays were produced from individualized clones of libraries prepared from *Pst*I-based genomic representations [44]. Two genomic representations comprising 11520 DArT markers were generated from 53 *Musa *genotypes ([45]). A random sample of 92 plants, out of the 180 analyzed with SSRs, was analyzed on each representation. The DArT markers heterozygous on one parent only were scored as co-dominant (segregation ratio 1:1), whereas the DArT markers polymorphic on both parents were scored as dominant (segregation ratio 1:3).

### Segregation distortion

As a diagnostic test for marker reliability, all markers were tested for significant deviation from expected Mendelian segregation ratios using a chi-square test prior to linkage. Markers that deviated significantly from Mendelian ratios were re-checked for scoring errors. Distorted markers are denoted on the map representations with asterisks according to their distortion level (i.e. 1* P < 0.05, 2* P < 0.01, 3* P < 0.005, 4* P < 0.001, 5* P < 0.0005, 6* P < 0.0001, 7* P < 0.00005).

### Linkage map construction

Borneo and P. Lilin parental maps were built from the analysis of marker segregation in the progeny. The P. Lilin map was further refined based on hypotheses of structural rearrangements. Finally the structure of each chromosome of structural heterozygous pairs was given on the basis of the structural pattern leading to a simulated NJ tree (see below) that fitted the observed one.

A third map, named the "synthetic map", is meant to represent the standard/ancestral *Musa *chromosomes without rearrangement events. It was designed in part by using consensus linkage groups of parental maps. For the remaining non-consensus groups, we kept as a skeleton the parental linkage group supposed to be free of any rearrangement. Then, markers of the other homolog parental linkage group were introduced one by one and mapped when they did not affect the previous order. The assessment of normal versus rearranged linkage group was essentially based on the absence of skewed markers, but also based on the linearity of a Neighbor-Joining (NJ) tree representation of the linkage groups (see below). The final reference map is an "artificial" map supposed to best represent the "standard" *Musa *genome.

The linkage groups of each parental map were defined using JoinMap^® ^4 software with a "cross-pollinated" population type [46]. Segregating DArTs markers present in the two sharing parents (dominant marker segregation type) were discarded, as they were poorly informative and not numerous in our data. A range of LOD scores from 3 to 8 was tested. The map was built at LOD 3.5 for Borneo, LOD 5 for P. Lilin and LOD 5 for the "synthetic map" using the regression mapping algorithm with the default parameters (i.e. a recombination frequency < 0.4, a mapping LOD score threshold of 1 and Kosambi mapping function [46]). When linkage groups of the parental maps displayed collinearity, consensus groups were established integrating all initial genotyping data of the progeny, with the default parameters. In case of discrepancy in the alignment of the two linkage groups, we imposed the marker order from one parent as a fixed one for the other one and vice-versa. If the fixed orders gave incoherent mapping (negative distances, high mean chi-square, suspect double recombinants...), each parental group was drawn up independently.

Group names were assigned according to a previous partial map [7] if common markers were found. New names were given otherwise.

### Graphic representation of linkage groups

Genetic mapping relies on the strong assumption that the recombination frequencies observed in a segregating population are accurate estimators of the genetic distances between markers physically aligned along the chromosomes. Therefore, defining the linear locus order and the distances between loci is strictly equivalent to a geometrical problem of adjusting the observed dissimilarities (the recombination frequencies) to a linear distance.

However, in case of heterozygous structural rearrangement, the observed recombination frequencies are locally a derivative of two unknown recombination frequencies corresponding to two different linear orders. For the affected loci, the conventional mapping methods propose a consensus that has no real biological meaning and they fail to map these loci correctly. However, keeping the geometrical problem analogy offers a means to localize these rearrangements graphically (Seguin, personal communication). Relaxing the constraint of linearity, the observed dissimilarities are adjusted to a so-called additive distance which is the distance on a tree-like graph [47] where the rearrangements appear as branches grafted on the basal line of unaffected loci. A linear distance is a particular case of additive tree distance, so in absence of rearrangement, the exhibited solution will be the optimal linear order of the loci. Several methods are available for adjusting a dissimilarity to a tree distance such as the NJ tree method [48], which remains computationally efficient even for a very large number of loci. These methods are based on agglomerative algorithms that join at the first step two close loci and progressively aggregate the closest remaining loci. This ascendant procedure means that the graph is essentially determined by the smallest dissimilarities. So, the highest values, between remote loci, which are truncated to 0.5 by construction in case of genetic, mapping, do not really disturb the tree construction

Keys to read the generated trees are given in Figure 3 from simulated data. A first set of chromosomes is generated with a marker each 5 cM. A second set is derived from the first one but with a particular rearrangement event. The simulated recombination frequencies are the arithmetic means of the genetic distance in the two chromosome sets. The simulation presumes that each segment of chromosome is able to pair with its homolog regardless of the structures of the chromosomes.

Figure 3-A is constructed from data of homologous chromosomes displaying fully aligned markers. It is the "normal" expected feature for homologous chromosomes. As soon as some rearrangements occur, the tree representation generates different figures such as Figure 3-B obtained in case of inversion. A distal non-reciprocal translocation generates a typical Y image (Figure 3-C), while a distal reciprocal translocation generates a typical X- shaped tree (Figure 3-D).

We applied the Weighted Neighbor-Joining algorithm implemented in DARwin 5 software [49] for the graphic representation of all the parental linkage groups identified under JoinMap^® ^4. For each linkage group independently, we exported from JoinMap^® ^4 the pair-wise recombination frequencies which were converted into map distances using the Kosambi mapping function. The distance matrix was then exported to DARwin5 for NJ tree calculation and for graphic representation of the linkage group.

## Competing interests

Andrzej Kilian (Director) and Eric Huttner are affiliated with Diversity Arrays Technology who provide DArT array commercial genotyping services for a range of crops.

## Authors' contributions

IH participated in the conception and design of the study, genomic SSR primers design, SSR genotyping, NJ and mapping analysis and paper writing; FB in the conception and design of the study, embryo rescue, cytogenetics, chromosome counting, paper writing; MS in theoretical and methodological development of NJ tree applied to genetic mapping and paper reviewing, LG and RR in SSR genotyping, AMR in the development of the SSR enriched library, DArT array construction and paper reviewing; CJ in the creation of the population and design of the study, XP in the NJ analysis (theory and simulation), paper writing, FC in the development of the SSR enriched library, XA in EST SSR primers' design, IAK in DNA extraction and genotyping, RNGM in DNA extraction; PP in the EST library construction; GJP and TM in the EST library sequencing, DM in EST sequences generation, VB in the SSR enriched library sequencing, EH and AK in the DArT genotyping, FCB, AD and FXC in the conception of the study, BC in mapping analysis and paper writing and JCG in the conception of the study and paper writing. All authors read and approved the final manuscript.

## Supplementary Material

Additional file 1 **Borneo genetic map built at LOD 3.5**. Molecular marker names are on the right side of each linkage group whereas genetic distances are on the left (cM; Kosambi mapping function). Loci labeled with asterisks showed distorted segregation (1* P < 0.05, 2* P < 0.01, 3* P < 0.005, 4* P < 0.001, 5* P < 0.0005, 6* P < 0.0001, 7* P < 0.00005). Anchor markers are underlined. In SSR names, mMaCIR has been abbreviated to CIR, and mMECIR to ECIR. Click here for file

Additional file 2 **Pisang Lilin genetic map built at LOD 5**. Molecular marker names are on the right side of each linkage group whereas genetic distances are on the left (cM; Kosambi mapping function). Loci labeled with asterisks showed distorted segregation (1* P < 0.05, 2* P < 0.01, 3* P < 0.005, 4* P < 0.001, 5* P < 0.0005, 6* P < 0.0001, 7* P < 0.00005). Anchor markers are underlined. In SSR names, mMaCIR has been abbreviated to CIR, and mMECIR to ECIR. Click here for file

Additional file 3 Neighbor-joining tree designed from linkage group 6+8 of Borneo defined at LOD 3.5; Markers in grey are DArTs markers, those in black SSR markers. Click here for file
