# Supplementary material for: A saturated SSR/DArT linkage map of Musa acuminata addressing genome rearrangements among bananas
Source: BMC Plant Biol. 2010 Apr 13;10:65. doi: 10.1186/1471-2229-10-65 (PMC2923539; doi:10.1186/1471-2229-10-65)
Supplement: Additional file 2 — Pisang Lilin genetic map built at LOD 5. Molecular marker names are on the right side of each linkage group whereas genetic distances are on the left (cM; Kosambi mapping function). Loci labeled with asterisks showed distorted segregation (1* P < 0.05, 2* P < 0.01, 3* P < 0.005, 4* P < 0.001, 5* P < 0.0005, 6* P < 0.0001, 7* P < 0.00005). Anchor markers are underlined. In SSR names, mMaCIR has been abbreviated to CIR, and mMECIR to ECIR. [file 1471-2229-10-65-S2.DOC]

Additional Figure 2
