# Supplementary material for: A saturated SSR/DArT linkage map of Musa acuminata addressing genome rearrangements among bananas
Source: BMC Plant Biol. 2010 Apr 13;10:65. doi: 10.1186/1471-2229-10-65 (PMC2923539; doi:10.1186/1471-2229-10-65)
Supplement: Additional file 3 — Neighbor-joining tree designed from linkage group 6+8 of Borneo defined at LOD 3.5; Markers in grey are DArTs markers, those in black SSR markers. [file 1471-2229-10-65-S3.DOC]

Additional Figure 3
